# Effectiveness of intra-articular injections of sodium bicarbonate and calcium gluconate in the treatment of osteoarthritis of the knee: a randomized double-blind clinical trial

**DOI:** 10.1186/s12891-015-0568-4

**Published:** 2015-05-13

**Authors:** Sandra García-Padilla, Miguel Angel Duarte-Vázquez, Karla Elena Gonzalez-Romero, María del Carmen Caamaño, Jorge L Rosado

**Affiliations:** Cindetec A.C, Parque Industrial Querétaro, Jurica 122, C.P. 76220 Santiago de Querétaro, Qro Mexico; Nucitec S.A. de C.V, Santiago de Querétaro, Mexico; Universidad Autónoma de Querétaro, Santiago de Querétaro, Mexico

**Keywords:** Osteoarthritis therapy, Joint, Knee, Sodium bicarbonate and calcium gluconate

## Abstract

**Background:**

A novel therapeutic management of osteoarthritis (OA) of the knee was assessed. The study aimed to evaluate the effect of monthly sodium bicarbonate with a single (SBCG1) or double dose (SBCG2) of calcium gluconate injections on OA of the knee; as well as the efficacy and safety of both SBCG interventions in the long term.

**Methods:**

A double-blind parallel-group clinical trial with 74 knee OA patients was performed during 12 months, both SBCG interventions were followed-up for another 6mo after intervention. The outcome variables were the Western Ontario-McMaster University Osteoarthritis Index (WOMAC), the Lequesne’s functional index and joint-space width changes from serial radiographs.

**Results:**

After 12 months, group SBCG1 decreased −14.8 (95% CI:-14.2, −17.0) and group SBCG2 decreased −14.6 (−16.9, −12.4) in the global WOMAC score, the mean changes represent 80% and 82% lessened pain, respectively. In the Lequesne Functional Index scale, SBCG1 decreased −11.9 (−10.4, −14.2) and SBCG2 decreased -11.9 (−13.8, −10.0), representing 66 and 69% of improvement. Both mean scores were maintained after intervention discontinued. SBCG2 improved the knees’ joint space width more than SBCG1 at 3 and 18 months. Both SBCG interventions were well tolerated after 12 months of treatment

**Conclusion:**

A solution of sodium bicarbonate and calcium gluconate is effective on reducing the symptoms associated with OA. Its beneficial effect is maintained for one year of continuous monthly administration and at least for 6 months after the administration is discontinued. When the dose of calcium gluconate is increased, it prevents further narrowing of joint-space.

**Trial registration:**

Clinicaltrials.gov NCT00977444 September 11, 2009.

## Background

Osteoarthritis (OA) is the most common joint disease; it is a consequence of cartilage degradation. OA may affect several joints, especially weight-bearing joints such as the knees. A joint’s cartilage degradation is clinically recognized by a gradual development of pain, stiffness, and loss of motion. Among the elderly, hip and knee OA causes a greater degree of disability [1]. It is estimated that 9.6% of men and 18% of women above 60 years have symptomatic OA primarily in the knees and hips [2].

Therapeutic management of OA of the knee has focused on pain relief, preserving or improving the range of motion and preventing secondary functional disability as well as joint damage. Besides surgery, which is recommended for severe cases, there are a wide variety of OA therapies commonly used: Non-pharmacological treatments are mainly aimed to unload the joint, such as weight loss or the use of lateral wedge insoles for medial OA of the knee; and pharmacological therapies which include analgesics, nonsteroidal anti-inflammatory drugs (NSAIDS), opioids, hyaluronic acid (HA) or corticosteroid injections and various drugs purported as disease-modifying osteoarthritis drugs (DMOADs) [3]. According to a recent review of knee OA therapeutic responses, HA injections, corticosteroids injections and opioids have demonstrated the highest effect of the recommended treatments [4]. However, none of these interventions have demonstrated to halt the disease progression. Moreover, several side effects have been demonstrated, mainly from oral NSAIDS treatments, which have been associated with gastrointestinal side effects [5], and opioids have been strongly associated with constipation, nausea, vomiting, dizziness, somnolence [6]. HA injections may lead to increase medial co-contraction and accelerate joint deterioration [7]. Corticosteroid injections, although they have not demonstrated to change the functionality of the knee, they are associated with reductions in knee pain over 2 weeks, however, these clinical improvements disappear by 4 weeks [8] and it is unsafe to inject it more than 4 times per year [9]. Thus, therapies appear to be misdirected and a different approach should be used to evaluate different alternatives.

Past research on OA treatments reported a beneficial effect of large doses of bicarbonate on certain chronic joint diseases; the effect was attributed to its alkalinity [10]. Previous research also found a beneficial effect of calcium gluconate on arthritis and other rheumatic diseases, this compound operates by allowing the linkage between chondrals and bone proteins, which avoids the hyperosmotic and acid conditions of the extracellular matrix and allows recovery of the homeostatic mechanisms of the cartilage [11]. Therefore a pharmaceutical solution of a combination of these two compounds might be beneficial to treat knee OA patients.

The present study aimed to evaluate the efficacy of an intra-articular administration of a sodium bicarbonate solution combined with two different concentrations of calcium gluconate injections on progression of symptoms and joint space width as well as the safety of these injections in patients with knee OA.

## Methods

### Patients

Patients eligible for the study were males and females aged ≥40 years with at least 1 year of being diagnosed with OA of the knee. According with the criteria of the American College of Rheumatology, OA was confirmed with physical exploration diagnosis of knee pain plus one of the following: bone crackling with movement, morning stiffness ≤15 min, age > 50 years or articular hypertrophy. And grade II-IV knee OA was confirmed by radiology according to the Kellgren-Lawrence grading system [12,13].

Patients diagnosed with grade I were referred for a less invasive treatment and patients with grade IV were included to evaluate the treatment effect in OA most advanced conditions. Three physicians examined and diagnosed the recruited patients, two were orthopedic specialist and one was surgeon specialized in radiology. Exclusion criteria included: An intra-articular injection of any substance administered within the last 3 months, joint inflammatory diseases, microcrystalline arthropathies, current pregnancy, uncontrolled hypertension, active infection, undergone surgery/arthroscopy within the last 3 months and diagnosis by radiography of knee OA Kellgren and Lawrence grade I, coagulation or platelet disorders or any concomitant disease that could interfere with the evaluation,. Patients were enrolled by public advertising and were studied at the San José Hospital in Queretaro, México.

The study was conducted in compliance with ICH (International Committee of Harmonization) Good Clinical Practices and the Declaration of Helsinki, and its applicable amendments. It was approved by the institutional review board for human research of the University of Querétaro and all subjects voluntarily signed informed consent before being enrolled in the study.

A total of 26 patients per experimental group were necessary to detect a clinically significant change of 20% within experimental treatments, from baseline to post treatment evaluations in the global score of Lequesne Index and Western Ontario and McMastern University Index (WOMAC) indexes considering an estimated standard deviation of 35%, a two-sided alpha level of 0.05 and a statistical power of 0.8. Considering a possible drop-out rate of 35%, 72 patients had to be recruited. In addition, the mentioned sample size can find a significant difference of 30% between two experimental groups’ changes in the global score of Lequesne and WOMAC. The parameters used to calculate the sample size were estimated from the results of a pilot study previously carried out with 18 participants.

### Study design

The study was a randomized, double-blind, parallel-design clinical trial. Patients were followed up during 12 months with treatments and for 6 months after intervention to evaluate a longer term effect and safety of the treatment. Subjects who met the selection criteria were randomly assigned to receive one of two treatments in both knees (except for 2 patients with prosthesis only one knee was intervened): 1) Sodium bicarbonate and calcium gluconate (SBCG1) 2) Sodium bicarbonate and a double dose of calcium gluconate (SBCG2). The randomization method was based on a list of randomly assorted treatments, generated by using an online computing program [14]. One researcher that had no direct contact with patients created the randomization list and delivered it to each physician that examined the patients who were assigned to each physician in a systematic order. The treatment was administered to each patient every 30 days by one of the 3 trained and experienced physicians who followed the same patients throughout the study. In the baseline evaluation and in the monthly scheduled visits during intervention period and post-intervention follow-up, patients were clinically evaluated with the WOMAC and Lequesne questionnaires and also monitored for adverse events. All the fieldwork personnel were blinded to the treatments.

Knee radiographs were taken to each patient at baseline, after 3 months, after 12 months of intervention and also after 6 months of post-intervention follow up. Patients were not permitted to receive concomitant treatment with analgesic or systemic corticosteroids.

### Treatments

The treatments were identical in packaging, labeling, schedule of administration and appearance. Both treatments, SBCG1 and SBCG2 were pharmaceutical compositions in aqueous solution (5 mL) ready for intra-articular infrapatellar injection. They were prepared at the pilot plant of the Center for Research and Technological Development in Chronic Disease (Cindetec A.C.). The SBCG1 treatment was a solution with sodium bicarbonate and calcium gluconate, both at a concentration of 7.5% (w/v); and SBCG2 had the same concentration of sodium bicarbonate than SBCG1, while the concentration of calcium gluconate was 15% (w/v).

### Efficacy assessments

The primary efficacy outcome variables were the changes from baseline to post-intervention assessments in the WOMAC, and Lequesne’s functional indexes and joint space width.

The WOMAC OA index is a validated, multidimensional, disease specific, health status measure. It provides with clinically important patient relevant symptoms in the areas of pain, stiffness and physical function in patients with hip and/or knee OA. It consists of 24 questions in three separated subscales: pain, physical function and stiffness. Each subscale score weighed 10 points, and the WOMAC global scale is the sum of the three subscales and ranges from 0 to 30 [15]. The Lequesne index is a 10-question interview-style survey that includes evaluation of pain or discomfort, maximum walking distance and daily activities performance. The total questionnaire is scored on a 24 points scale [16].

Joint space width changes were assessed on radiographs that followed standardized techniques [17]. At each time point 4 knee radiographs were obtained per patient, to get from each knee a standing anterior-posterior and lateral views. All radiographs were taken by the same technician and were interpreted by 2 independent physicians who were blinded to treatment, both with certifications in orthopedics and traumatology. The outcome measure was the progression of joint space narrowing as suggested by Abadie et al. [18]. One independent collaborator, blinded to treatment measured joint space width in radiographs by visual reading according to a validated method [19] at the joint’s narrowest point using an X10 magnifying lens graduated at 0.1 mm intervals.

### Safety evaluation

The safety of experimental treatments was assessed with reported adverse events, defined as any unfavorable and unintended sign, symptom, or disease temporary present during the intervention period, whether or not related to the treatment. Adverse events were either reported by the patient or discovered by physicians during visits. Adverse events were classified into light, moderate, serious and lethal according to the Mexican Official Norm for Drug Surveillance [20]. In addition patients were monitored with laboratory tests (hematology, biochemistry and urine analysis) at baseline, 12 and 18 months.

### Statistical analysis

Baseline demographic variables and treatments’ compliance were analyzed with the chi square test. The collected data were analyzed in an intention-to-treat basis with subjects that received the allocated intervention and attended to at least one post-treatment evaluation. The WOMAC, and Lequesnes´s functional indexes’ mean values were calculated for each evaluation and a Student *T* test was used to evaluate the significance of changes at each post-treatment evaluation compared with baseline evaluation. Mean changes between both treatments at each evaluation were compared with ANOVA.

In the radiographies' analysis, the narrowest joint space from both measurements in each knee was selected for analysis. Mean values were calculated for both knees joint spaces and a paired *T*-test was performed to evaluate joint space change for each treatment. To evaluate the response between treatments, a Generalized Estimating Equation model was performed to evaluate joint space changes, this model considered the correlation between each subject’s knees and the baseline values as a covariate.

To evaluate safety of the treatment, adverse events were classified by seriousness and were quantified to get the incidence of each adverse event.

All statistical models were tested at the 0.05 level of significance. Analyses were performed with SPSS® for Windows version 18.0 (IBM®).

## Results

**Figure 1:**
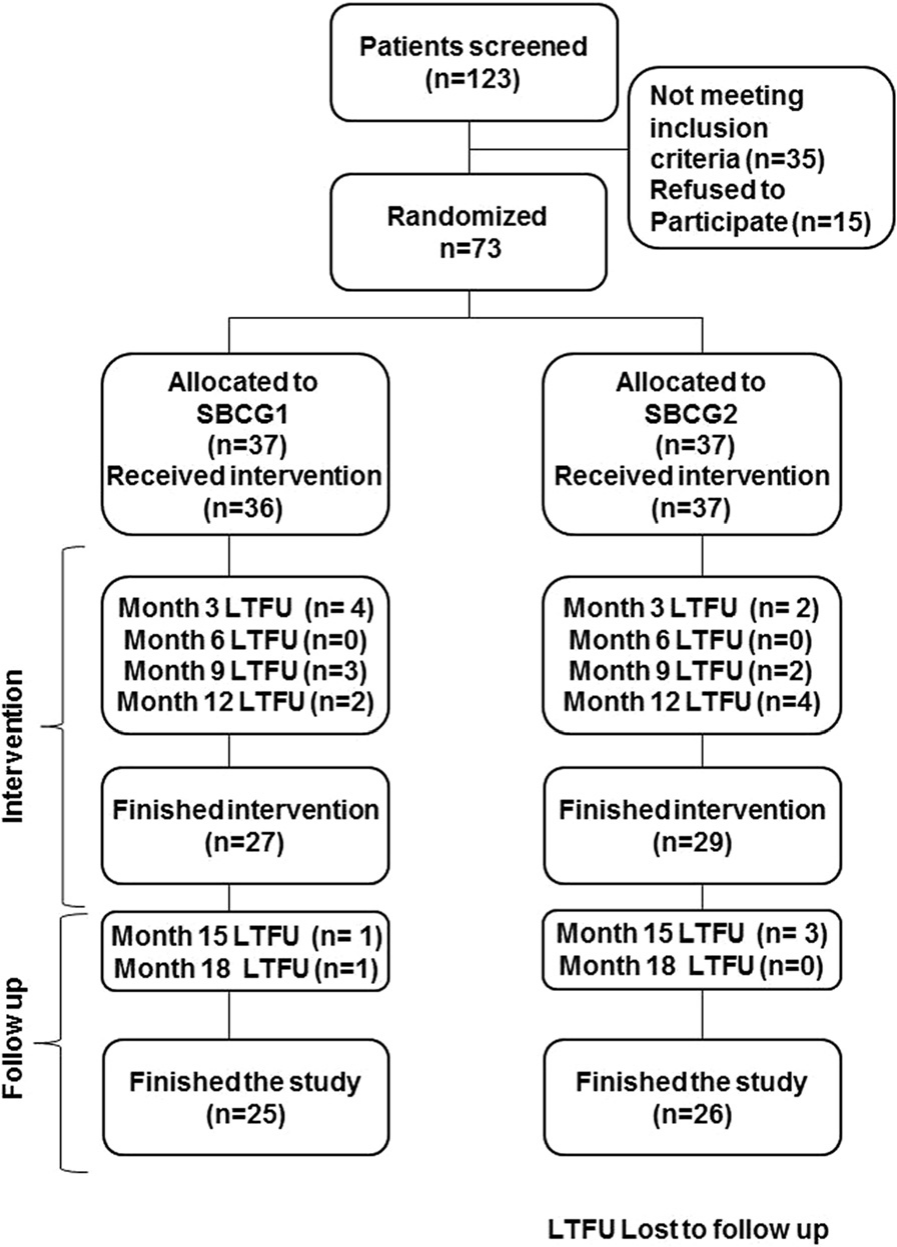
Flow of patients through the study.

A total of 123 patients were initially screened for the study between December 2007 and February 2008, 50 did not meet the inclusion criteria or refused to participate. A total of 74 subjects were randomly assigned to one of the two treatment groups, one refused to receive the allocated intervention, 21 refused to continue at some point during the trial due to personal reasons and 1 due to a rash that according with the physician should not continue with the treatment. Thus, 51 patients finished the intervention and follow up period (Figure 1).

The baseline demographic characteristics of the patients that initiated the study and the patients that completed the study did not differ among the experimental groups (Table 1). The majority of patients were female (61/73), mean ± SD age was 54.9 ± 9.3 years. The body mass index mean was 31.6 ± 4.7 Kg/m^2^. Baseline radiographic analysis showed no significant difference among treatment groups in joint-space narrowing and marginal osteophytes formation, within each knee compartment. In the majority of the patients (59/73), pain intensity in the target knee was reported from moderate to severe.

**Table 1:** **Baseline demographic characteristics of patients with osteoarthritis of the knee, by study group** ^**a**^

**Variables**	**Baseline sample**			**Final sample**		
	**SBCG1**	**SBCG2**	**Sig.** ^**b**^	**SBCG1**	**SBCG2**	**Sig.** ^**b**^
N	36	37		25	26	
Age, y	55.22 ± 9.75	54.59 ± 8.98	0.775	54.44 ± 9.34	53.81 ± 8.61	0.802
BMI kg/cm^2^	31.14 ± 4.86	31.98 ± 4.57	0.453	31.66 ± 5.24	32.44 ± 4.62	0.579
Female	88.9	78.4	0.226	92.0	80.8	0.244
Location and grade* of OA						
Left knee: Grade II	19.4	19.4	0.858	24.0	24.0	1.000
Grade III	50.0	55.6		48.0	48.0	
Grade IV	30.6	25.0		28.0	28.0	
Right knee: Grade II	19.4	16.2	0.843	24.0	19.2	0.893
Grade III	50.0	56.8		48.0	53.8	
Grade IV	30.6	27.0		28.0	26.9	

^a^Values are % or mean ± SD.

^b^ANOVA or Chi Square significance level of group comparisons.

*According to Kellgren-Lawrence classification of OA.

After one year in the study 27 and 29 subjects from SBCG1 and SBCG2, respectively, finished the intervention period (Figure 1); all of them received the allocated intervention. Most of the drop outs (85%) were voluntary for personal reasons, which are expected due to the long duration of the study. The statistical power with the subjects that finished the study was above 90%.

**Figure 2:**
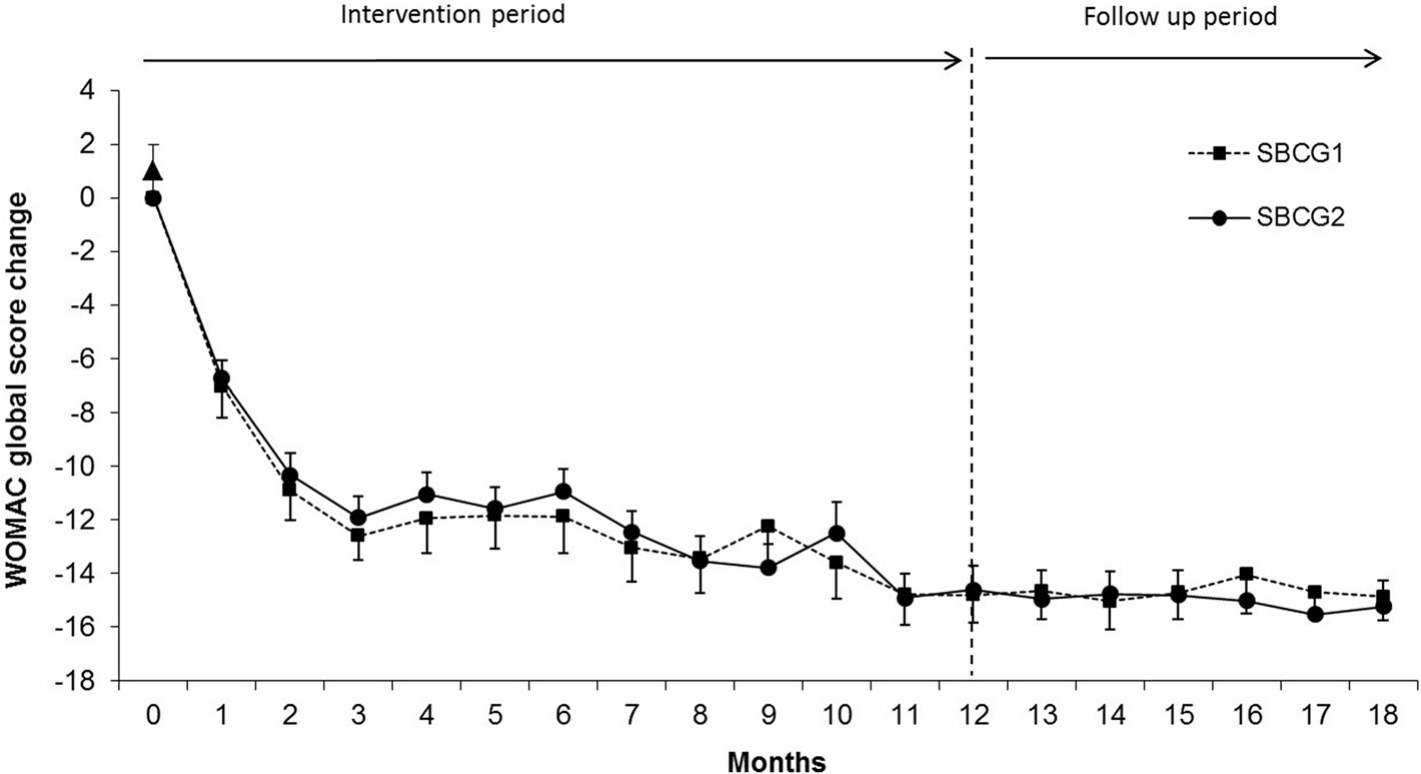
Monthly changes in WOMAC pain index by treatment group. Means (±SEM) are adjusted for baseline values. All post-treatment measurements were significantly different from baseline value. There were no significant differences between treatments.

Baseline and unadjusted as well as adjusted mean changes of the WOMAC subscales and global scores at different time points until 18 months are shown in Table 2. At the end of the intervention, patients in SBCG1 and SBCG2 groups, showed a significant improvement compared with their baseline values in all WOMAC subscales: 81% and 77% in pain, 92% and 79% in stiffness and 90% and 81% in physical functioning, respectively. The changes in WOMAC total score along the period of treatment are shown in Figure 2. The mean score that decreased at 12 months was maintained 6 months after treatment suspension. There was no difference within treatments during intervention or during post-treatment period.

**Table 2:** **Evaluation of the experimental formulation during 12 months of intervention and 6 months of follow up in WOMAC subscales**

**WOMAC subscales**	**SBCG1**		**SBCG2**	
	**Score**	**Change from baseline**	**Score**	**Change from baseline**
Pain				
Baseline	5.8 (5.2, 6.4) ^a^		5.6 (5.0, 6.2)	
3 mo.	1.9 (1.1, 2.8) ^b^	−4.0 (−7.0, −5.1)	2.4 (1.7, 3.2)	−3.2 (−3.9, −2.5)
8 mo.	1.6 (1.0, 2.2)	−4.2 (−9.1, −5.1)	1.5 (1.0, 2.0)	−4.0 (−4.9, −3.2)
12 mo.	1.1 (0.6, 1.7)	−4.5 (−10.5, −5.3)	1.3 (0.8, 1.9)	−4.5 (−5.2, −3.8)
18 mo.	1.2 (0.5, 1.9)	−4.3 (−8.4, −5.3)	1.0 (0.5, 1.5)	−4.8 (−5.6, −4.0)
Stiffness				
Baseline	5.9 (5.0, 6.7)		6.3 (5.6, 7.0)	
3 mo.	2.0 (1.3, 2.6)	−4.2 (−9.0, −5.1)	2.1 (1.3, 2.9)	−4.0 (−4.9, −3.2)
8 mo.	1.0 (0.5, 1.5)	−4.8 (−9.3, −5.8)	1.5 (0.9, 2.0)	−4.8 (−5.7, −3.8)
12 mo.	0.5 (0.2, 0.9)	−5.2 (−10.7, −6.2)	1.3 (0.6, 2.0)	−4.9 (−5.9, −4.0)
18 mo.	0.7 (0.0, 1.4)	−5.0 (−7.9, −6.3)	1.1 (0.4, 1.7)	−5.0 (−6.0, −4.0)
Physical functioning				
Baseline	5.9 (5.2, 6.6)		6.2 (5.4, 6.9)	
3 mo.	2.1 (1.5, 2.8)	−3.8 (−7.6, −4.9)	2.1 (1.5, 2.8)	−3.8 (−4.5, −3.1)
8 mo.	1.4 (0.9, 1.9)	−4.6 (−10.2, −5.5)	1.5 (1.1, 2.0)	−4.7 (−5.6, −3.9)
12 mo.	0.6 (0.4, 0.9)	−5.1 (−13.3, −5.9)	1.2 (0.6, 1.7)	−5.2 (−6.1, −4.3)
18 mo.	0.7 (0.3, 1.2)	−4.8 (−9.6, −5.9)	1.0 (0.5, 1.4)	−5.2 (−6.2, −4.3)
Global score				
Baseline	17.5 (15.7, 19.3)		18.1 (16.3, 19.9)	
3 mo.	6.0 (4.2, 7.9)	−11.9 (−9.1, −14.7)	6.7 (4.7, 8.7)	−11.1 (−13.1, −9.1)
8 mo.	4.2 (2.7, 5.6)	−13.5 (−10.4, −16.1)	4.5 (3.1, 5.9)	−13.6 (−15.9, −11.2)
12 mo.	2.3 (1.5, 3.1)	−14.8 (−14.2, −17.0)	3.8 (2.1, 5.5)	−14.6 (−16.9, −12.4)
18 mo.	2.6 (0.8, 4.4)	−14.0 (−9.7, −17.1)	3.1 (1.5, 4.6)	−15.0 (−17.5, −12.6)

^a^All values are means (95% confidence interval).

^b^All post-treatment measurements were significantly different from baseline value. There were no significant differences between treatments.

**Figure 3:**
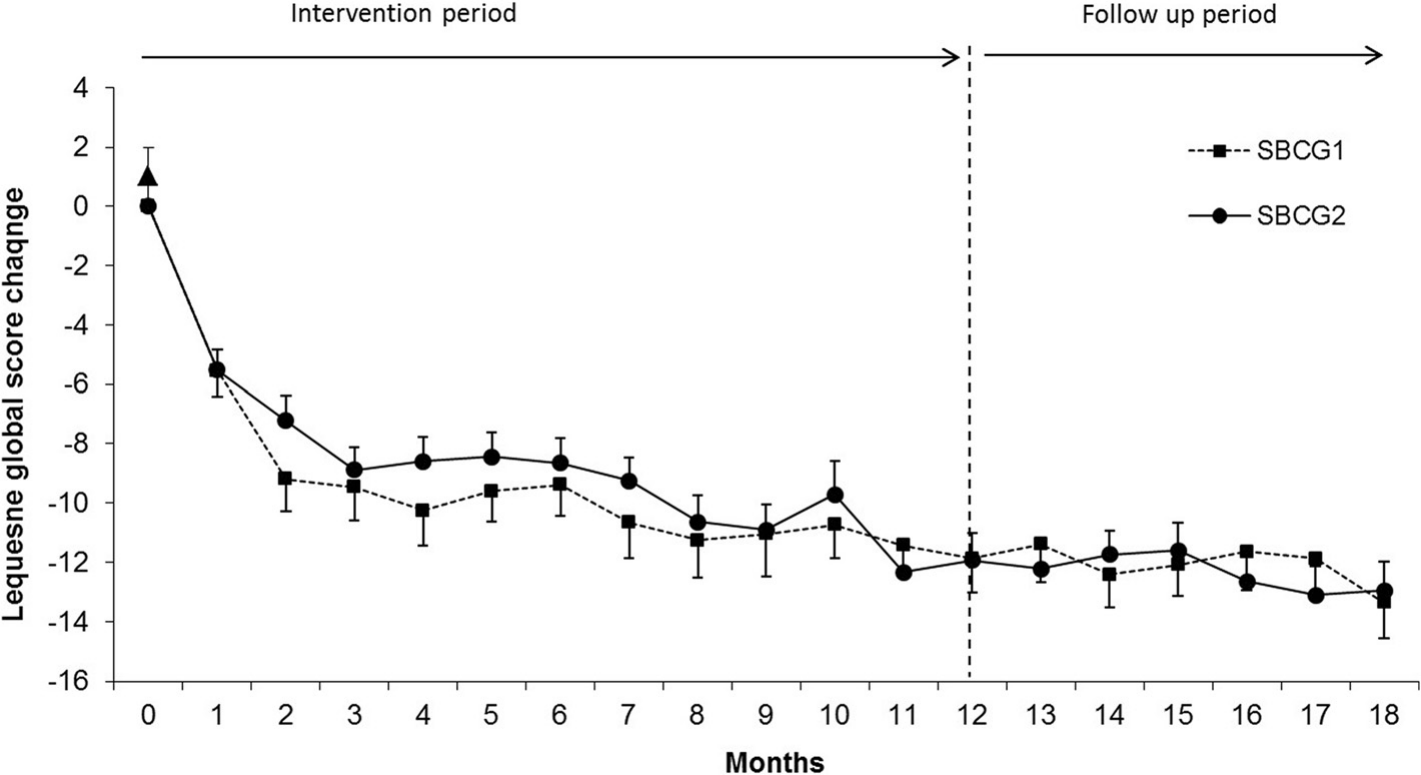
Monthly changes in Lequesne´s functional index by treatment group. Means (±SEM) are adjusted for baseline values. All post-treatment measurements were significantly different from baseline value. There were no significant differences between treatments.

Baseline values and unadjusted and adjusted mean changes of the Lequesne functional index subscales and global scores at different time points until 18 months are shown in Table 3. After 12 months of treatment, patients in both groups showed a significant improvement in all score subscales, for SBCG1 and SBCG2 groups, there was a 74% and 69% reduction in pain, 74% and 71% improvement in maximum walking distance and 65% and 56% improvement in the daily activities subscale, respectively. Figure 3 shows the changes in Lequesne´s functional Index along the study period. During the first 3 months of intervention the Lequesne´s functional index score decreased substantially and from month 4 to month 12 the improvement was maintained. After intervention period there was a reduction in the Lequene´s total score of about 68% and 69% with SBCG1 and SBCG2 groups, respectively, compared with baseline values. There was no difference within treatments during intervention and post-intervention period.

**Table 3:** **Evaluation of the experimental formulation during 12 months of intervention and 6 months of follow up in Lequesne Functional Index subscales**

**Lequesne subscales**	**SBCG1**		**SBCG2**	
	**Score**	**Change from baseline**	**Score**	**Change from baseline**
Pain				
Baseline	5.4 (4.9, 5.9)^a^	-	5.5 (5.1, 5.9)	-
3 mo.	2.7 (2.1, 3.3)^b^	−3.0 (−7.5, −3.8)	2.4 (1.8, 3.1)	−2.9 (−3.6, −2.2)
8 mo.	2.0 (1.5, 2.6)	−3.4 (−9.0, −4.1)	1.9 (1.3, 2.6)	−3.5 (−4.3, −2.8)
12 mo.	1.4 (1.0, 1.9)	−3.8 (−9.9, −4.6)	1.7 (1.1, 2.4)	−3.9 (−4.6, −3.3)
18 mo.	1.3 (0.7, 1.9)	−3.8 (−8.8, −4.7)	1.7 (1.0, 2.4)	−4.0 (−4.7, −3.4)
Maximum walking distance				
Baseline	4.2 (3.4, 5.1)	-	4.2 (3.6, 4.8)	-
3 mo.	1.8 (0.9, 2.6)	−2.9 (−6.4, −3.8)	2.2 (1.5, 2.9)	−1.9 (−2.7, −1.2)
8 mo.	1.2 (0.5, 1.9)	−3.2 (−6.8, −4.2)	1.4 (0.8, 1.9)	−2.9 (−3.7, −2.1)
12 mo.	1.1 (0.4, 1.8)	−3.2 (−6.7, −4.2)	1.2 (0.6, 1.8)	−3.3 (−4.1, −2.5)
18 mo.	0.7 (−0.1, 1.4)	−3.3 (−6.0, −4.4)	0.7 (0.2, 1.2)	−3.7 (−4.4, −2.9)
Daily activities				
Baseline	8.0 (6.8, 9.2)	-	7.5 (6.7, 8.3)	-
3 mo.	4.0 (3.3, 4.8)	−4.4 (−5.7, −6.0)	3.9 (3.3, 4.6)	−3.7 (−4.6, −2.8)
8 mo.	3.5 (2.9, 4.2)	−4.7 (−6.3, −6.2)	3.5 (2.9, 4.1)	−4.2 (−5.2, −3.2)
12 mo.	2.8 (2.2, 3.4)	−4.9 (−7.8, −6.2)	3.3 (2.6, 4.0)	−4.7 (−5.8, −3.6)
18 mo.	2.8 (2.1, 3.6)	−4.5 (−6.3, −6.0)	2.9 (2.2, 3.7)	−5.0 (−6.4, −3.5)
Global score				
Baseline	17.6 (15.5, 19.8)	-	17.2 (15.9, 18.5)	-
3 mo.	8.5 (6.7, 10.2)	−10.3 (−8.6, −12.7)	8.6 (7.0, 10.2)	−8.6 (−10.2, −6.9)
8 mo.	6.8 (5.2, 8.3)	−11.3 (−8.7, −13.9)	6.8 (5.3, 8.3)	−10.6 (−12.5, −8.8)
12 mo.	5.3 (3.8, 6.8)	−11.9 (−10.4, −14.2)	6.2 (4.6, 7.8)	−11.9 (−13.8, −10.0)
18 mo.	4.8 (3.1, 6.4)	−11.6 (−8.9, −14.4)	5.3 (3.7, 6.9)	−12.7 (−14.8, −10.5)

^a^All values are means (95% confidence interval).

^b^All post-treatment measurements were significantly different from baseline value.

There were no significant differences between treatments.

**Figure 4:**
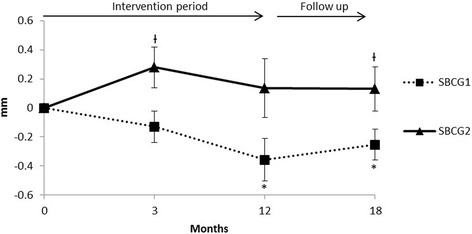
Joint space width changes in patients completing the intervention and 6 months follow-up. Errors bars represent SEM. *Different to baseline value in Paired *T*-test (p < 0.05). ^Ɨ^Significantly different from SBCG2 in a Generalized Estimating Equation model that considered the correlation between both knees from each subject, and also considered the baseline evaluation values as a covariate.

Joint space narrowing, within the knee joint during treatment with intra-articular administration of SBCG1 and SBCG2 is shown in Figure 4. After the 12 month-period of treatment there was a significant decrease in joint-space width of −0.37 (95% CI: −0.64, −0.10) mm in SBCG1 group, and there was no significant change in SBCG2 group: 0.15 (−0.33, 0.63) mm. The mean joint space change of SBCG2 was significantly higher than SBCG1 group after 4 and 18 months.

The percentage of patients who experienced any adverse event, during this study did not differ between both groups (32% and 37% for SBCG1 and SBCG2, respectively). The majority of adverse events (77%) were related to knee OA: knee pain, nuisance or weakness, stiffness, burning and numbness. The rest of the events were not related with the knee or the intervention: respiratory or gastrointestinal infections, headache and lumbar pain among others. None of the reported adverse events was classified as severe. The laboratory tests during all evaluations did not indicate abnormal values in patients.

## Discussion

The purpose of this study was to evaluate the effectiveness of a combination of sodium bicarbonate and calcium gluconate at physiological concentrations of 1:1 (Wt/Vol) (SBCG1) and a concentration of 1:2 (Wt/Vol) (SBCG2) administrated directly into the knee joint, on OA symptoms and joint space width, as well as the safety of the experimental treatments. The baseline values of the WOMAC index of patients involved in this study corresponded to symptoms of mild to moderate severity.

The study confirmed that both SBCG formulations improved OA symptoms during and after a period of 12 months and that the effect was maintained for 6 additional months without treatment. These improvements exceeded those that have been considered clinically significant [21-24]. According to a review of Raynauld et al. [25], intra-articular injection of hyaluronate reduced knee pain in patients with OA by 20-40% over 6–12 months. In another randomized, placebo controlled trial, patients taking 1500 mg oral glucosamine sulfate daily for three years showed significant reduction of 34.1% in total WOMAC index compared with baseline values [26]. In the present study, both treatment groups experienced a reduction on the WOMAC global scale of 84% which is a substantial functional improvement.

Similarly, both experimental groups improved the pain, maximum walking distance and daily activities scores according to Lequesne´s functional index. A reduction of about 30% in Lequesne´s functional index has been defined as an effective treatment [27]. In a 3-year, randomized double-blind study, a treatment with glucosamine sulfate produced a reduction of about 20% in the Lequesne´s index total score [28]. In another study, five weekly intra-articular injections of hyaluronate produced a reduction of 45% after 6 months (from 10.3 ± 3.7 points at baseline to 5.7 ± 3.2 points after treatment) [29]. In the present study, the mean improvement in the Lequesne´s global score after 12 months was of 70 and 64% for SBCG1 and SBCG2 groups, respectively. These changes are above the values used to define treatment effectiveness and more effective than previous studies. No further changes were observed during the 6 months follow-up period which shows that the benefits of the administration of this solution will remain for at least six months.

The effect of the experimental treatments on joint space width, was evaluated by measuring the change in width of medial tibio-femoral joints’ space since it is the primary outcome recommended by several authors [17,30,31]. After one year of treatment, a slight narrowing of joint-space width was observed in the SBCG1 group. The SBCG2 group did not experience a narrowing in joint space during the intervention period. Several studies have assessed the natural narrowing of joint space width in patients with OA, it has been observed a joint-space width change of about −0.6 mm/year [32]. A large long-term study has shown that a joint space narrowing of no more than −0.1 mm/year could bring clinical benefits [33]. The mean joint space narrowing in the SBCG1 group was above that value (−0.37 mm), this suggests that although SBCG1 relieves symptoms of OA, the lower concentration of calcium gluconate does not stop its natural progression. In contrast, administration of SBCG2 prevented this naturally occurring joint space narrowing (0.36 mm). This effect was evident after the first year of treatment and remained after 6 months follow-up with no treatment. Therefore, a higher dose of calcium gluconate may decelerate joint space narrowing or even widen the joint space. Calcium gluconate may produce a similar or greater effect than glucosamine sulfate, a compound that has proved to modify the joint structure, since several reported clinical trials have shown a little change in joint-space width (~ −0.06 mm/year) with glucosamine sulfate [26,34]. Therefore, the effect of calcium gluconate on joint-space narrowing found in the present study could be even more beneficial than glucosamine sulfate in a longer term. Moreover, according to Abadie et al. [18] SBCG2 may have the characteristics to become a DMOADs. Thus, further research needs to be performed to confirm the effect of the experimental treatment in joint space narrowing compared with other potential pharmacological drugs.

The experimental treatment was developed upon previous works on the beneficial effect of large doses of bicarbonate on certain chronic joint diseases because of its alkalinity. Schweiz et al. [10] administered intra-articular isotonic sodium bicarbonate 1-29% in several doses from 0.5 to 2.0 mL at 2-day intervals. Two to four injections were administered depending on each patient’s symptoms improvement. The knee-joint was the most often and most successfully treated. Additionally, Beckett in a US patent [35], described the use of an aqueous neutral to mildly alkaline bicarbonate solution useful for preventing and treating inflammatory diseases such as OA. It has been documented in *in-vitro* studies that sodium bicarbonate activates the Na^+^-dependent Cl^−^ -HCO_3_^−^ exchanger which promotes a recovery of the intracellular pH of chondrocyte leading a normalization of intracellular metabolic activities [36]; and as a result, a rearrangement of the extracellular matrix is expected [37-39].

The effect of calcium gluconate in the treatment of inflammatory diseases was also reported previously. Walter in a US patent [11] described the use of corticosteroid combined with calcium gluconate for arthritis and other rheumatic diseases that involved an inflammatory process. Recently, Kang et al. demonstrated that calcium gluconate has a protective effect against OA through inhibition of COX-2 and related chondrocytes apptosis [40]. Calcium compounds applied simultaneously with corticosteroids in the treatment of rheumatic diseases, reduced inflammation. It has also been reported that calcium gluconate allows the linkage between chondrals and bone proteins, which avoids the hyperosmotic and acid conditions of the extracellular matrix and allows recovery of the homeostatic mechanisms of the cartilage [41].

The mechanism of action of the SBCG formulation has not been fully elucidated yet. The effect of sodium bicarbonate and calcium gluconate over clinical symptoms of OA and joint space could be due to its effects on cartilage metabolism, including stimulation of anabolic activities, such as the synthesis of proteoglycans, and the depression of catabolic activities such as the effects of caspase-3 and Poly-ADP ribose polymerase (PARP) a key mediators of apoptosis of chondrocytes.

To clarify the precise mechanism by which SBCG formulation produces a beneficial effect on knee OA, further studies with longer follow-up period and different designs are needed. Although significant therapeutic effects of intra-articular injection of SBCG for knee OA were observed in this study, the following limitations must be considered: 1) Since recruited patients attended to the hospital to get an OA treatment, it was not appropriate to provide a placebo treatment, thus all patients received treatment. 2) There was no active-control treatment, a review of results from similar studies that use the same evaluation scales was considered enough to evaluate the treatment’s effect compared with other treatment options. 3) The sample included a wide range of OA severity and segmented analyses based on the severity of OA were not possible to perform due to lack of statistical power. 4) Another limitation of the study was that radiographic views were used to assess joint structural changes; it is known that magnetic resonance imaging is more accurate; however significant differences were found with the increased variability that radiographs could cause. The findings in this study are applicable to Hispanic adults with knee OA that are seeking for a treatment.

## Conclusion

The findings of the present study demonstrate that a combination of sodium bicarbonate and calcium gluconate administered once a month directly into the knee joint produces a highly significant reduction of pain and improvement in physical function measured with WOMAC total score and Lequesne´s functional index starting from the first intervention. The experimental formulation‘s improvement is maximized until 10 to 12 months; and its beneficial effect is maintained for at least 6 months after the administration is discontinued. Only when the dose of calcium gluconate is increased, it eliminates further narrowing of joint space. Although the possible mechanism is not known at present, the administration of these two physiological substances in combination represents a highly effective and safe alternative for the treatment of knee OA.

## Abbreviations

OA: Osteoarthritis
HA: Hyaluronic acid
NSAIDs: Non-steroidal anti-inflammatory drugs
WOMAC: Western Ontario-McMaster University Osteoarthritis Index
SBCG: Sodium bicarbonate and calcium gluconate
