# Erratum to: Effectiveness of intra-articular injections of sodium bicarbonate and calcium gluconate in the treatment of osteoarthritis of the knee: a randomized double-blind clinical trial

**DOI:** 10.1186/s12891-015-0673-4

**Published:** 2015-09-17

**Authors:** Sandra García-Padilla, Miguel Angel Duarte-Vázquez, Karla Elena Gonzalez-Romero, María del Carmen Caamaño, Jorge L Rosado

**Affiliations:** Cindetec A.C, Parque Industrial Querétaro, Jurica 122, Santiago de Querétaro, C.P. 76220 Qro Mexico; Nucitec S.A. de C.V, Santiago de Querétaro, Mexico; Universidad Autónoma de Querétaro, Santiago de Querétaro, Mexico

## Erratum

The original version of this article [1] unfortunately contained a mistake. The formula concentration described in the “Treatments” section was provided incorrectly. The corrected text is available below:

“The SBCG1 treatment was a solution with sodium bicarbonate and calcium gluconate, both at a concentration of 0.75 % (w/v); and SBCG2 had the same concentration of sodium bicarbonate than SBCG1, while the concentration of calcium gluconate was 1.5 % (w/v).
